# Reattack of a Certificateless Aggregate Signature Scheme with Constant Pairing Computations

**DOI:** 10.1155/2014/343715

**Published:** 2014-03-13

**Authors:** Hang Tu, Debiao He, Baojun Huang

**Affiliations:** ^1^School of Computer, Wuhan University, Wuhan 430072, China; ^2^School of Mathematics and Statistics, Wuhan University, Wuhan 430072, China

## Abstract

A new attack against a novel certificateless aggregate signature scheme with constant pairing computations is presented. To enhance security, a new certificateless signature scheme is proposed first. Then a new certificateless aggregate signature scheme with constant pairing computations based on the new certificateless signature scheme is presented. Security analysis shows that the proposed certificateless aggregate signature scheme is provably secured in the random oracle.

## 1. Introduction

The concept of aggregate signature (AS) scheme was first introduced by Boneh et al. [1] in 2003. Such a scheme greatly reduces the computational and communication overhead since it could aggregate *n* signatures on *n* distinct messages from *n* distinct users into a single signature and check the correctness through a verification operation. The AS scheme is very useful in real-world applications. For example, in the scenario of the secure Border Gateway Protocol (BGP) [2], each router successively signs its own segment of a path in the network and then forwards the collection of signatures associated with the path to the next router. The AS scheme can be used to compress these signatures into a single one and hence reduce the overheads of both bandwidth and computation required in the original secure BGP. Similarly, in the scenario of the Vehicular Ad Hoc Networks (VANETs) [3], each vehicle or roadside unit (RSU) has to verify around 500–2000 messages per second. The AS scheme can be used to compress these messages into a single one and check the correctness through a verification operation. To satisfy different applications, many AS schemes based on the traditional public key cryptography (TPKC) and identity-based public key cryptography (ID-based PKC) have been proposed.

It is well known that a certificate, generated by a trusted third party, is needed to bind a user's identity and its public key in the TPKC. However, the management of these certificates becomes more and more difficult with the growth of users' number. To solve the problem, Shamir [4] introduced the concept of the ID-based PKC. In such cryptography, the user's identity, such as name, email address, and telephone number, is his public key and his private key is generated by the key generation centre (KGC) using his identity. However, the key escrow problem exists in the ID-based PKC since the KGC know the user's private key. In 2003, Al-Riyami and Paterson [5] developed the concept of the CLPKC to solve the key escrow problem in the ID-PKC. In CLPKC, the KGC only generates a partial private key for a user and the full private key of the user is a combination of his partial private key and some secret value chosen by the user himself. Recently, CLPKC attracted much attention and many certificateless encryption (CLE) schemes [6–8], certificateless key agreement (CLKA) schemes [9–11], certificateless signcryption (CLSC) schemes [12, 13], and certificateless signature (CLS) schemes [14–16] were proposed.

To satisfy the applications in certificateless environment, the certificateless aggregate signature (CLAS) scheme has attracted much attention. Several CLAS schemes [17–23] have been proposed by different researchers. However, most of these schemes [17–20, 23] have computational complexity for pairing computations that grows linearly with the number of signers, which deviates from the main goals of aggregate signatures. Besides, both of the schemes [20, 22] of Zhang et al. require certain synchronization; that is, all signers must share the same synchronized clocks to generate aggregate signature. It is easy to say that it is difficult to achieve synchronization in many communication scenarios. Shim [24] pointed out that L. Zhang and F. Zhang's scheme [20] is vulnerable to the coalition attack. Xiong et al. [25] found that Hu et al.'s scheme [21] cannot provide unforgeability. Very recently, Xiong et al. [25] proposed a certificateless signature (CLS) scheme and constructed a new CLAS scheme using that CLS scheme. Compared to previous CLAS schemes, Xiong et al.'s CLAS scheme is very efficient in computation, and the verification procedure needs only a very small constant number of pairing operations, independent of the number of aggregated signatures. Besides, their scheme does not require synchronization for aggregating randomness. Unfortunately, He et al. [26] pointed out that Xiong et al.'s CLAS scheme is insecure against a Type II adversary by giving concrete attack. However, He et al. did not give countermeasure to enhance security. In this paper, we propose a new attack against Xiong et al.'s CLAS scheme; that is, a Type II adversary could forge legal signature for any message. To improve security, we also propose an improved CLAS scheme.

The organization of the paper is sketched as follows.  Section 2 gives some preliminaries of the paper. Sections 3 and 4 review and analyze Xiong et al.'s scheme.  Section 5 gives our improved scheme. Sections 6 and 7 discuss security and performance analysis of our scheme. At last, we give some conclusion in Section 8.

## 2. Preliminaries

### 2.1. Bilinear Pairing

Let *G*_1_ be a cyclic additive group of prime order *q* and *G*_2_ a cyclic multiplicative group of the same order *q*. We let *P* denote the generator of *G*_1_. A bilinear pairing is a map *e* : *G*_1_ × *G*_1_ → *G*_2_ which satisfies the following properties.

(1) Bilinearity
  (1) $$\begin{array}{c} e\left(aQ,bR\right)=e{\left(Q,R\right)}^{ab}, \end{array}$$

where *Q*, *R* ∈ *G*_1_, *a*, *b* ∈ *Z*_q_*.

(2) Nondegeneracy
  (2) $$\begin{array}{c} e\left(P,P\right)\neq 1_{G_{2}}. \end{array}$$
(3) Computability: there is an efficient algorithm to compute *e*(*Q*, *R*) for all *Q*, *R* ∈ *G*_1_.

The Weil and Tate pairings associated with supersingular elliptic curves or abelian varieties can be modified to create such admissible pairings. The following problems are assumed to be intractable within polynomial time.

*Computational Diffie-Hellman (CDH) Problem.* Given *aP*, *bP* ∈ *G*_1_, the task of CDH problem is to compute *a* *bP*, where *P* denotes the generator of *G*_1_.

### 2.2. Formal Model of CLS and CLAS

In this subsection, we will review the definition and security notions specified in [25], only with slight notational differences. There are two kinds of adversaries in the CLS scheme and the CLAS scheme, that is, the Type I adversary *𝒜*1 and the Type II adversary *𝒜*2. The adversary *𝒜*1 is not able to access the master key but he could replace public keys at his will. The adversary *𝒜*2 represents a malicious KGC who generates partial private key of users. *𝒜*2 could have access to the master key of KGC, but he is not able to replace public keys. The following are five oracles which can be accessed by the adversaries.

- *CreateUser*: Given an identity *ID*_*i*_, if *ID*_*i*_ has already been created, nothing is to be carried out. Otherwise, the oracle generates the partial private key *psk*_*ID*_*i*__, the secret key *usk*_*ID*_*i*__, and the public key *upk*_*ID*_*i*__. It then stores the tuple (*ID*_*i*_, *psk*_*ID*_*i*__, *upk*_*ID*_*i*__, *usk*_*ID*_*i*__) into a list *L*. In both cases, *psk*_*ID*_*i*__ is returned.
- *RevealPartialKey*: On input of an identity *ID*_*i*_, the oracle searches *L* for a corresponding entry to *ID*_*i*_. If it is not found, ⊥ is returned; otherwise, the corresponding *psk*_*ID*_*i*__ is returned.
- *RevealSecertKey*: On input of an identity *ID*_*i*_, the oracle searches *L* for a corresponding entry to *ID*_*i*_. If it is not found, ⊥ is returned; otherwise, the corresponding *usk*_*ID*_*i*__ is returned.
- *ReplaceKey*: On input of an identity *ID*_*i*_ and a user public/secret key pair (*upk*_*ID*_*i*__′, *usk*_*ID*_*i*__′), the oracle searches *L* for the entry of *ID*_*i*_. If it is not found, nothing will be carried out. Otherwise, the oracle updates (*ID*_*i*_, *psk*_*ID*_*i*__, *upk*_*ID*_*i*__, *usk*_*ID*_*i*__) to (*ID*_*i*_, *psk*_*ID*_*i*__, *upk*_*ID*_*i*__′, *usk*_*ID*_*i*__′).
- *Sign*: On input of a message *m*_*i*_ for *ID*_*i*_, the signing oracle proceeds in one of the three cases below.
  - A valid signature *σ*_*i*_ returned if *ID*_*i*_ has been created but the user public/secret key pair (*usk*_*ID*_*i*__, *upk*_*ID*_*i*__) has not been replaced.
  - If *ID*_*i*_ has not been created, a symbol ⊥ is returned.
  - If the user public/secret key pair of *ID*_*i*_ has been replaced with say (*upk*_*ID*_*i*__′, *usk*_*ID*_*i*__′), then the oracle returns the result of *Sign*(*usk*_*ID*_*i*__′, *psk*_*ID*_*i*__′, *m*_*i*_).

The security for a CLS scheme and a CLAS scheme is defined via the following two games separately.

**Game 1**

The first game is performed between a challenger *𝒞* and an adversary *𝒜* ∈ {*𝒜*1, *𝒜*2} for a CLS scheme as follows.

- *𝒞* executes* MasterKeyGen* to get master private/public key pair (*mpk*, *msk*).
- *𝒜* can adaptively issue the* CreateUser*,* RevealPartialKey*,* RevealSecertKey*,* ReplaceKey*, and *Sign* queries to *𝒞*.
- *𝒜* is to output a message *m*_i_* and a signature *σ*_i_* corresponding to a target identity *ID*_i_* and a public key *upk*_*ID*_i_*_.

We say that *𝒜* wins Game 1, if and only if the following three conditions hold.

1. *σ*_i_* is a valid signature on messages *m*_i_* under identities *ID*_i_* and the corresponding public key *upk*_*ID*_i_*_.
2. If *𝒜* is *𝒜*1, the identity *ID*_i_* has not submitted to* RevealPartialKey* queries to get the partial private key *psk*_*ID*_i_*_. If *𝒜* is *𝒜*2, *ID*_i_* has not submitted to* RevealSecertKey* queries or* ReplaceKey* queries to get the secret key *usk*_*ID*_i_*_.
3. The oracle *Sign* has never been queried with (*ID*_i_*, *m*_i_*).

**Definition 1**

A CLS scheme is said to be secure if there is no probabilistic polynomial-time adversary *𝒜* ∈ {*𝒜*1, *𝒜*2}, which wins Game 1 with nonnegligible advantage.

**Game 2**

The second game is performed between a challenger *𝒞* and an adversary *𝒜* ∈ {*𝒜*1, *𝒜*2} for a CLAS scheme as follows.

- *𝒞* executes* MasterKeyGen* to get master private/public key pair (*mpk*, *msk*).
- *𝒜* can adaptively issue the* CreateUser*,* RevealPartialKey*,* RevealSecertKey*,* ReplaceKey*, and* Sign* queries to *𝒞*.
- *𝒜* outputs a set of *n* users whose identities are from the set *L*_ID_* = {*ID*_1_*,…, *ID*_n_*} and corresponding public keys from the set *L*_upk_* = {*upk*_1_*,…, *upk*_n_*}, *n* messages *L*_m_* = {*m*_1_*,…, *m*_n_*}, and an aggregate signature σ*.

We say that *𝒜* wins Game 2, if and only if the following three conditions hold.

1. *σ*_i_* is a valid aggregate signature on messages {*m*_1_*,…, *m*_n_*} under identities {*ID*_1_*,…, *ID*_n_*} and the corresponding public key {*upk*_1_*,…, *upk*_n_*}.
2. If *𝒜* is *𝒜*1, at least one of the identities *ID*_i_* has not submitted to* RevealPartialKey* queries to get the partial private key *psk*_*ID*_i_*_. If *𝒜* is *𝒜*2, at least one of *ID*_i_* has not been submitted to* RevealSecertKey* queries or* ReplaceKey* queries to get the secret key *usk*_*ID*_i_*_.
3. The oracle* Sign* has never been queried with (*ID*_i_*, *m*_i_*).

**Definition 2**

A CLAS scheme is said to be secure if there is no probabilistic polynomial-time adversary *𝒜* ∈ {*𝒜*1, *𝒜*2}, which wins Game 2 with nonnegligible advantage.

## 3. Review of Xiong et al.'s Schemes

### 3.1. Xiong et al.'s CLS Scheme

In this subsection, we will briefly review Xiong et al.'s CLS scheme. Their CLS scheme consists of five algorithms:* MasterKeyGen*,* PartialKeyGen, UserKeyGen, Sign*, and* Verify*. The detail of these algorithms is described as follows.

*MasterKeyGen.* Given a security parameter *k*, KGC runs the algorithm as follows.

1. Generate a cyclic additive group *G*_1_ and a cyclic multiplicative group *G*_2_ with prime order *q*.
2. Generate two generators *P*, *Q* of *G*_1_ and an admissible pairing *e* : *G*_1_ × *G*_1_ → *G*_2_.
3. Generate a random number *s* ∈ *Z*_q_* and compute *P*_*pub*_ = *sP*.
4. Choose cryptographic hash functions *H*_1_ : {0,1}* → *G*_1_ and *H*_2_ : {0,1}* → *Z*_q_*.
5. KGC publishes the system parameters {*q*, *G*_1_, *G*_2_, *e*, *P*, *Q*, *P*_*pub*_, *H*_1_, *H*_2_} and key the master key *s* secretly.

*PartialKeyGen*. Given a user's identity *ID*_*i*_, KGC computes the user's partial private key *psk*_*ID*_*i*__ = *sQ*_*ID*_*i*__ and transmits it to the user secretly, where *Q*_*ID*_*i*__ = *H*_1_(*ID*_*i*_). 

*UserKeyGen*. The user with identity *ID*_*i*_ selects a random number *x*_*ID*_*i*__ ∈ *Z*_q_* as his secret key *usk*_*ID*_*i*__ and computes his public key as *upk*_*ID*_*i*__ = *usk*_*ID*_*i*__ · *P*. 

*Sign*. Given a message *m*_*i*_, the partial private key *psk*_*ID*_*i*__, the secret key *usk*_*ID*_*i*__, the user with identity *ID*_*i*_, and the corresponding public key *upk*_*ID*_*i*__, perform the following steps to generate a signature.

1. Generate a random number *r*_*i*_ ∈ *Z*_q_* and compute *U*_*i*_ = *r*_*i*_ *P*.
2. Compute *h*_*i*_ = *H*_2_(*m*_*i*_, *ID*_*i*_, *upk*_*ID*_*i*__, *U*_*i*_), *V*_*i*_ = *psk*_*ID*_*i*__ + *h*_*i*_ · *r*_*i*_ · *P*_*pub*_ + *h*_*i*_ · *x*_*ID*_*i*__ · *Q*.
3. Output (*U*_*i*_, *V*_*i*_) as the signature on *m*_*i*_.

*Verify*. Given a signature (*U*_*i*_, *V*_*i*_) of message *m*_*i*_ on identity *ID*_*i*_ and corresponding public key *upk*_*ID*_*i*__:

1. Compute *Q*_*ID*_*i*__ = *H*_1_(*ID*_*i*_) and *h*_*i*_ = *H*_2_(*m*_*i*_, *ID*_*i*_, *upk*_*ID*_*i*__, *U*_*i*_).
2. Verify *e*(*V*_*i*_, *P*) = *e*(*h*_*i*_ · *U*_*i*_ + *Q*_*ID*_*i*__, *P*_*pub*_)*e*(*h*_*i*_ · *upk*_*ID*_*i*__, *Q*) holds or not. If it holds, accept the signature.

### 3.2. Xiong et al.'s CLAS Scheme

In this subsection, we will briefly review Xiong et al.'s CLAS scheme. Their CLAS scheme consists of six algorithms:* MasterKeyGen, PartialKeyGen, UserKeyGen, Sign, Aggregate*, and* AggregateVerify*. The first four algorithms are the same as those in their CLS scheme. The detail of the other two algorithms is described as follows.

*Aggregate*. For an aggregating set of *n* users {*𝒰*_1_,…, *𝒰*_*n*_} with identities {*ID*_1_,…, *ID*_*n*_}, corresponding public keys {*upk*_1_,…, *upk*_*n*_}, and message-signature pairs {(*m*_1_, *σ*_1_ = (*U*_1_, *V*_1_)),…, (*m*_*n*_, *σ*_*n*_ = (*U*_*n*_, *V*_*n*_))} from {*𝒰*_1_,…, *𝒰*_*n*_}, respectively, the aggregate signature generator computes *V* = ∑_*i*=1_^*n*^ *V*_*i*_ and outputs *σ* = (*U*_1_,…, *U*_2_, *V*) as an aggregate signature.

*AggregateVerify*. To verify an aggregate signature *σ* = (*U*_1_,…, *U*_2_, *V*) signed by *n* users {*𝒰*_1_,…, *𝒰*_*n*_} with identities {*ID*_1_,…, *ID*_*n*_} and the corresponding public keys {*upk*_1_,…, *upk*_*n*_} on messages {*m*_1_,…, *m*_*n*_}, the verifier performs the following steps.

1. Compute *Q*_*ID*_*i*__ = *H*_1_(*ID*_*i*_) and *h*_*i*_ = *H*_2_(*m*_*i*_, *ID*_*i*_, *upk*_*ID*_*i*__, *U*_*i*_) for *i* = 1,…, *n*.
2. Verify *e*(*V*, *P*) = *e*(∑_*i*=1_^*n*^(*h*_*i*_ · *U*_*i*_ + *Q*_*ID*_*i*__), *P*_*pub*_)*e*(∑_*i*=1_^*n*^ *h*_*i*_ · *upk*_*ID*_*i*__, *Q*) holds or not. If it holds, accept the signature.

## 4. Cryptanalysis of Xiong et al.'s Scheme

Shim [24] claimed that both of their schemes are provably secure against two types of adversary in the random oracle model. However, in this section, we will disprove their claims by giving two concrete attacks.

### 4.1. Attack against Xiong et al.'s CLS Scheme

Shim [24] claimed their CLS scheme is semantically secure against Type II adversary. Unfortunately, it is not true, since there is a polynomial time Type II adversary *𝒜*2 who can always win Game 1 through either of the following two attacks.

In Xiong et al.'s CLS scheme, the system parameters {*q*, *G*_1_, *G*_2_, *e*, *P*, *Q*, *P*_*pub*_, *H*_1_, *H*_2_} are generated by KGC. Let a Type II adversary *𝒜*2 be a malicious KGC. Then *𝒜*2 could choose a random *t* and compute *Q* = *tP*, when he generates the system parameters. After that, *𝒜*2 could forge a legal signature of any message.

1. The Type II adversary *𝒜*2 has the master key *s*. Then, he could compute a user *𝒰*_*i*_'s partial private key *psk*_*ID*_*i*__ = *sQ*_*ID*_*i*__, where *Q*_*ID*_*i*__ = *H*_1_(*ID*_*i*_).
2. For any message *m*_*i*_, *𝒜*2 generates a random number *r*_*i*_ ∈ *Z*_q_* and computes *U*_*i*_ = *r*_*i*_ *P*, *h*_*i*_ = *H*_2_(*m*_*i*_, *ID*_*i*_, *upk*_*ID*_*i*__, *U*_*i*_) and *V*_*i*_ = *psk*_*ID*_*i*__ + *h*_*i*_ · *r*_*i*_ · *P*_*pub*_ + *h*_*i*_ · *t* · *upk*_*ID*_*i*__.
3. *𝒜*2 outputs (*U*_*i*_, *V*_*i*_) as the signature on *m*_*i*_.

Since *Q* = *tP*, *U*_*i*_ = *r*_*i*_ *P*, and *V*_*i*_ = *psk*_*ID*_*i*__ + *h*_*i*_ · *r*_*i*_ · *P*_*pub*_ + *h*_*i*_ · *t* · *upk*_*ID*_*i*__, we could have

(3) e(Vi,P)=e(Vi=pskIDi+hi·ri·Ppub+hi·t·upkIDi,P)=e(pskIDi+hi·ri·Ppub,P)e(hi·t·upkIDi,P)=e(sQIDi+hi·ri·sP,P)e(hi·upkIDi,t·P)=e(hi·Ui+QIDi,sP)e(hi·upkIDi,Q)=e(hi·Ui+QIDi,Ppub)e(hi·upkIDi,Q).

Then, we know that (*U*_*i*_, *V*_*i*_) is a legal signature on *m*_*i*_. Besides, *ID*_*i*_ has not been submitted to* RevealSecertKey* queries or* ReplaceKey* queries to get the secret key *usk*_*ID*_i_*_ and the oracle *Sign* has never been queried with (*ID*_*i*_, *m*_*i*_). So the Type II adversary *𝒜*2 wins Game 1.

Therefore, Xiong et al.'s CLS scheme is not secure against attacks of the Type II adversary.

### 4.2. Attack against Xiong et al.'s CLAS Scheme

Shim [24] claimed their CLAS scheme is semantically secure against Type II adversary. Unfortunately, it is not true, since there exists a polynomial time Type II adversary *𝒜*2, who can always win Game 2 through either of the following two attacks.

In Xiong et al.'s CLAS scheme, the system parameters {*q*, *G*_1_, *G*_2_, *e*, *P*, *Q*, *P*_*pub*_, *H*_1_, *H*_2_} are generated by KGC. Let a Type II adversary *𝒜*2 be a malicious KGC. Then *𝒜*2 could choose a random *t* and compute *Q* = *tP*, when he generates the system parameters. After that, *𝒜*2 could forge an aggregate signature.

Let {*𝒰*_1_,…, *𝒰*_*n*_} be an aggregating set of *n* users with identities {*ID*_1_,…, *ID*_*n*_} and the corresponding public keys {*upk*_1_,…, *upk*_*n*_}.

1. For *i* = 1,2,…, *n*, *𝒜*2 does the following five substeps to generate a legal signature (*U*_*i*_, *V*_*i*_) on a message *m*_*i*_.
  - The Type II adversary *𝒜*2 has the master key *s*. Then, he could compute a user *𝒰*_*i*_'s partial private key *psk*_*ID*_*i*__ = *sQ*_*ID*_*i*__, where *Q*_*ID*_*i*__ = *H*_1_(*ID*_*i*_).
  - For any message *m*_*i*_, *𝒜*2 generates a random number *r*_*i*_ ∈ *Z*_q_* and computes *U*_*i*_ = *r*_*i*_ *P*, *h*_*i*_ = *H*_2_(*m*_*i*_, *ID*_*i*_, *upk*_*ID*_*i*__, *U*_*i*_), and *V*_*i*_ = *psk*_*ID*_*i*__ + *h*_*i*_ · *r*_*i*_ · *P*_*pub*_ + *h*_*i*_ · *t* · *upk*_*ID*_*i*__.
  - *𝒜*2 outputs (*U*_*i*_, *V*_*i*_) as the signature on *m*_*i*_.
2. *𝒜*2 computes *V* = ∑_*i*=1_^*n*^ *V*_*i*_.
3. *𝒜*2 outputs *σ* = (*U*_1_,…, *U*_*n*_, *V*) as an aggregate signature.

From the analysis in the above subsection, we know that (*U*_*i*_, *V*_*i*_) satisfies the equation *e*(*V*_*i*_, *P*) = *e*(*h*_*i*_ · *U*_*i*_ + *Q*_*ID*_*i*__, *sP*_*pub*_)*e*(*h*_*i*_ · *upk*_*ID*_*i*__, *Q*), where *Q* = *tP* and *V*_*i*_ = *psk*_*ID*_*i*__ + *h*_*i*_ · *r*_*i*_ · *P*_*pub*_ + *h*_*i*_ · *t* · *upk*_*ID*_*i*__. Then we could have that

(4) e(V,P)=e(∑i=1nVi,P)=e(∑i=1n(pskIDi+hi·ri·Ppub+hi·t·upkIDi),P)=e(∑i=1n(pskIDi+hi·ri·Ppub),P) ×e(∑i=1nhi·t·upkIDi,P)=e(∑i=1n(hi·Ui+QIDi),Ppub) ×e(∑i=1nhi·upkIDi,Q).

Thus, we know that *σ* = (*U*_1_,…, *U*_*n*_, *V*) is a legal aggregate signature on messages {*m*_1_,…, *m*_*n*_}. Besides, for any *i* ∈ {1,…, *n*}, *ID*_*i*_ has not been submitted to* RevealSecertKey* queries or* ReplaceKey* queries to get the secret key *usk*_*ID*_i_*_ and the oracle* Sign* has never been queried with (*ID*_*i*_, *m*_*i*_). So the Type II adversary *𝒜*2 wins Game 2.

Therefore, Xiong et al.'s CLAS scheme is not secure against attacks of the Type II adversary.

## 5. Our CLS Scheme and CLAS Scheme

### 5.1. Our CLS Scheme

Like Xiong et al.'s CLS scheme does, our CLS scheme also consists of five algorithms:* MasterKeyGen, PartialKeyGen, UserKeyGen, Sign*, and* Verify*. The detail of these algorithms is described as follows.

*MasterKeyGen*. Given a security parameter *k*, KGC runs the algorithm as follows.

1. Generate a cyclic additive group *G*_1_ and a cyclic multiplicative group *G*_2_ with prime order *q*.
2. Generate a generator *P* of *G*_1_ and an admissible pairing *e* : *G*_1_ × *G*_1_ → *G*_2_.
3. Generate a random number *s* ∈ *Z*_q_* and compute *P*_*pub*_ = *sP*.
4. Choose cryptographic hash functions *H*_1_, *H*_2_, *H*_3_ : {0,1}* → *G*_1_ and *H*_4_, *H*_5_ : {0,1}* → *Z*_q_*.
5. KGC publishes the system parameters *params* = {*q*, *G*_1_, *G*_2_, *e*, *P*, *P*_*pub*_, *H*_1_, *H*_2_, *H*_3_, *H*_4_, *H*_5_} and key the master key *s* secretly.

*PartialKeyGen*. Given a user's identity *ID*_*i*_, KGC computes the user's partial private key *psk*_*ID*_*i*__ = *sQ*_*ID*_*i*__ and transmits it to the user secretly, where *Q*_*ID*_*i*__ = *H*_1_(*ID*_*i*_). 

*UserKeyGen*. The user with identity *ID*_*i*_ selects a random number *x*_*ID*_*i*__ ∈ *Z*_q_* as his secret key *usk*_*ID*_*i*__ and computes his public key as *upk*_*ID*_*i*__ = *usk*_*ID*_*i*__ · *P*. 

*Sign*. Given a message *m*_*i*_, the partial private key *psk*_*ID*_*i*__, the secret key *usk*_*ID*_*i*__, the user with identity *ID*_*i*_, and the corresponding public key *upk*_*ID*_*i*__, perform the following steps to generate a signature.

1. Generate a random number *r*_*i*_ ∈ *Z*_q_* and compute *U*_*i*_ = *r*_*i*_ *P*.
2. Compute *W* = *H*_2_(*params*), *T* = *H*_3_(*params*), *h*_*i*_ = *H*_4_(*m*_*i*_, *ID*_*i*_, *upk*_*ID*_*i*__, *U*_*i*_), and *k*_*i*_ = *H*_5_(*m*_*i*_, *ID*_*i*_, *upk*_*ID*_*i*__, *U*_*i*_).
3. Compute, *V*_*i*_ = *psk*_*ID*_*i*__ + *h*_*i*_ · *x*_*ID*_*i*__ · *W* + *k*_*i*_ · *r*_*i*_ · *T*.
4. Output (*U*_*i*_, *V*_*i*_) as the signature on *m*_*i*_.

*Verify*. Given a signature (*U*_*i*_, *V*_*i*_) of message *m*_*i*_ on identity *ID*_*i*_ and corresponding public key *upk*_*ID*_*i*__, consider the following.

1. Compute *Q*_*ID*_*i*__ = *H*_1_(*ID*_*i*_), *W* = *H*_2_(*params*), *T* = *H*_3_(*params*), *h*_*i*_ = *H*_4_(*m*_*i*_, *ID*_*i*_, *upk*_*ID*_*i*__, *U*_*i*_), and *k*_*i*_ = *H*_5_(*m*_*i*_, *ID*_*i*_, *upk*_*ID*_*i*__, *U*_*i*_).
2. Verify *e*(*V*_*i*_, *P*) = *e*(*Q*_*ID*_*i*__, *P*_*pub*_)*e*(*h*_*i*_ · *upk*_*ID*_*i*__, *W*)*e*(*k*_*i*_ *U*_*i*_, *T*) holds or not. If it holds, accept the signature.

### 5.2. Our CLAS Scheme

Like Xiong et al.'s CLAS scheme does, our CLAS scheme also consists of six algorithms:* MasterKeyGen, PartialKeyGen, UserKeyGen, Sign, Aggregate*, and* AggregateVerify*. The first four algorithms are the same as those in our CLS scheme. The detail of other two algorithms is described as follows.

*Aggregate*. For an aggregating set of *n* users {*𝒰*_1_,…, *𝒰*_*n*_} with identities {*ID*_1_,…, *ID*_*n*_}, corresponding public keys {*upk*_1_,…, *upk*_*n*_}, and message-signature pairs {(*m*_1_, *σ*_1_ = (*U*_1_, *V*_1_)),…, (*m*_*n*_, *σ*_*n*_ = (*U*_*n*_, *V*_*n*_))} from {*𝒰*_1_,…, *𝒰*_*n*_}, respectively, the aggregate signature generator computes *V* = ∑_*i*=1_^*n*^ *V*_*i*_ and outputs *σ* = (*U*_1_,…, *U*_*n*_, *V*) as an aggregate signature.

*AggregateVerify*. To verify an aggregate signature *σ* = (*U*_1_,…, *U*_*n*_, *V*) signed by *n* users {*𝒰*_1_,…, *𝒰*_*n*_} with identities {*ID*_1_,…, *ID*_*n*_} and the corresponding public keys {*upk*_1_,…, *upk*_*n*_} on messages {*m*_1_,…, *m*_*n*_}, the verifier performs the following steps.

1. Compute *Q*_*ID*_*i*__ = *H*_1_(*ID*_*i*_), *W* = *H*_2_(*params*), *T* = *H*_3_(*params*), *h*_*i*_ = *H*_4_(*m*_*i*_, *ID*_*i*_, *upk*_*ID*_*i*__, *U*_*i*_), and *k*_*i*_ = *H*_5_(*m*_*i*_, *ID*_*i*_, *upk*_*ID*_*i*__, *U*_*i*_) for *i* = 1,…, *n*.
2. Verify *e*(*V*, *P*) = *e*(∑_*i*=1_^*n*^ *Q*_*ID*_*i*__, *P*_*pub*_)*e*(∑_*i*=1_^*n*^ *h*_*i*_ · *upk*_*ID*_*i*__, *Q*)*e*(∑_*i*=1_^*n*^ *k*_*i*_·*U*_*i*_, *T*) holds or not. If it holds, accept the signature.

## 6. Security Analysis

### 6.1. Security Analysis of Our CLS Scheme

In this section, we analyze the security of our CLS scheme. The following lemmas and theorem are proposed.

**Lemma 3**

If Type I adversary *𝒜*1 wins Game 1 with nonnegligible probability *ε*, then one could construct an algorithm to solve the CDH problem in *G*_1_ with nonnegligible probability.

**Proof**

Given an instance (*X* = *xP*, *Y* = *yP*) of the CDH problem in *G*_1_, we will construct an algorithm *𝒞* to compute *x* *yP*, where *x*, *y* ∈ *Z*_q_* and they are unknown to *𝒞*. At first, *𝒞* picks an identity *ID*_*I*_ at random as the challenged identity in this game, sets the master public key *P*_*pub*_ = *X*, selects the system parameters *params* = {*G*_1_, *G*_2_, *e*, *P*, *P*_*pub*_, *H*_1_, *H*_2_, *H*_3_, *H*_4_, *H*_5_}, and returns the parameters to *𝒜*1. Then *𝒞* answers *𝒜*1's query as follows.   

- *H*_1_ *query*: *𝒞* maintains a list *L*_*H*_1__ of form 〈*ID*_*i*_, *a*_*ID*_*i*__, *Q*_*ID*_*i*__〉. When *𝒜*1 makes this query on *ID*_*i*_, *𝒞* does as follows.
  - If the list *L*_*H*_1__ contains a tuple 〈*ID*_*i*_, *a*_*ID*_*i*__, *Q*_*ID*_*i*__〉, *𝒞* returns *Q*_*ID*_*i*__ to *𝒜*1.
  - Otherwise, if *ID*_*i*_ = *ID*_*I*_, *𝒞* picks a random *a*_*ID*_*i*__ ∈ *Z*_n_*, computes *Q*_*ID*_*i*__ = *a*_*ID*_*i*__ *Y*, adds 〈*ID*_*i*_, *a*_*ID*_*i*__, *Q*_*ID*_*i*__〉 to *L*_*H*_1__, and returns *Q*_*ID*_*i*__ to *𝒜*1.
  - Otherwise (*ID*_*i*_ ≠ *ID*_*I*_), *𝒞* picks a random *a*_*ID*_*i*__ ∈ *Z*_n_*, computes *Q*_*ID*_*i*__ = *a*_*ID*_*i*__ *P*, adds 〈*ID*_*i*_, *a*_*ID*_*i*__, *Q*_*ID*_*i*__〉 to *L*_*H*_1__, and returns *Q*_*ID*_*i*__ to *𝒜*1.
- *H*_2_ *query*: *𝒞* maintains a list *L*_*H*_2__ of form 〈*m*_*i*_, *b*_*i*_, *W*_*i*_〉. When *𝒜*1 makes this query on *m*_*i*_, *𝒞* does as follows.
  - If the list *L*_*H*_2__ contains a tuple 〈*m*_*i*_, *b*_*i*_, *W*_*i*_〉, *𝒞* returns *W*_*i*_ to *𝒜*1.
  - Otherwise, *𝒞* picks a random *b*_*i*_ ∈ *Z*_n_*, computes *W*_*i*_ = *b*_*i*_ *P*, adds 〈*m*_*i*_, *b*_*i*_, *W*_*i*_〉 to *L*_*H*_2__, and returns *W*_*i*_ to *𝒜*1.
- *H*_3_ *query*: *𝒞* maintains a list *L*_*H*_3__ of form 〈*m*_*i*_, *c*_*i*_, *T*_*i*_〉. When *𝒜*1 makes this query on *m*_*i*_, *𝒞* does as follows.  
  - If the list *L*_*H*_3__ contains a tuple 〈*m*_*i*_, *c*_*i*_, *T*_*i*_〉, *𝒞* returns *T*_*i*_ to *𝒜*1.
  - Otherwise, *𝒞* picks a random *c*_*i*_ ∈ *Z*_n_*, computes *T*_*i*_ = *c*_*i*_ *X*, adds 〈*m*_*i*_, *c*_*i*_, *T*_*i*_〉 to *L*_*H*_3__, and returns *T*_*i*_ to *𝒜*1.
- *H*_4_ *query*: *𝒞* maintains a list *L*_*H*_4__ of form 〈*m*_*i*_, *ID*_*i*_, *upk*_*ID*_*i*__, *U*_*i*_, *h*_*i*_〉. When *𝒜*1 makes this query on (*m*_*i*_, *ID*_*i*_, *upk*_*ID*_*i*__, *U*_*i*_), *𝒞* does as follows.  
  - If the list *L*_*H*_4__ contains a tuple 〈*m*_*i*_, *ID*_*i*_, *upk*_*ID*_*i*__, *U*_*i*_, *h*_*i*_〉, *𝒞* returns *h*_*i*_ to *𝒜*1.
  - Otherwise, *𝒞* picks a random *h*_*i*_ ∈ *Z*_n_*, returns *h*_*i*_ to *𝒜*1, and adds 〈*m*_*i*_, *ID*_*i*_, *upk*_*ID*_*i*__, *U*_*i*_, *h*_*i*_〉 to *L*_*H*_4__.
- *H*_5_ *query*: *𝒞* maintains a list *L*_*H*_5__ of form 〈*m*_*i*_, *ID*_*i*_, *upk*_*ID*_*i*__, *U*_*i*_, *k*_*i*_〉. When *𝒜*1 makes this query on (*m*_*i*_, *ID*_*i*_, *upk*_*ID*_*i*__, *U*_*i*_), *𝒞* does as follows.
  - If the list *L*_*H*_5__ contains a tuple 〈*m*_*i*_, *ID*_*i*_, *upk*_*ID*_*i*__, *U*_*i*_, *k*_*i*_〉, *𝒞* returns *k*_*i*_ to *𝒜*1.
  - Otherwise, *𝒞* picks a random *k*_*i*_ ∈ *Z*_n_*, returns *k*_*i*_ to *𝒜*1, and adds 〈*m*_*i*_, *ID*_*i*_, *upk*_*ID*_*i*__, *U*_*i*_, *h*_*i*_〉 to *L*_*H*_5__.
- * CreateUser*: *𝒞* maintains a list *L*_*U*_ of form 〈*ID*_*i*_, *psk*_*ID*_*i*__, *Q*_*ID*_*i*__, *usk*_*ID*_*i*__, *upk*_*ID*_*i*__〉. When *𝒜*1 makes this query on *ID*_*i*_, *𝒞* does as follows.
  - If the list *L*_*U*_ contains a tuple 〈*ID*_*i*_, *psk*_*ID*_*i*__, *Q*_*ID*_*i*__, *usk*_*ID*_*i*__, *upk*_*ID*_*i*__〉, *𝒞* returns *upk*_*ID*_*i*__ to *𝒜*1.
  - Otherwise, if *ID*_*i*_ = *ID*_*I*_, *𝒞* gets *Q*_*ID*_*I*__ = *a*_*ID*_*I*__ *Y* by making *H*_1_ query with *ID*_*I*_ and sets *psk*_*ID*_*I*__ ← ⊥. *𝒞* selects a random number *x*_*ID*_*i*__ ∈ *Z*_q_* as his secret key *usk*_*ID*_*i*__ and computes his public key as *upk*_*ID*_*i*__ = *usk*_*ID*_*i*__ · *P*. At last, *𝒞* adds 〈*ID*_*i*_, *psk*_*ID*_*i*__, *Q*_*ID*_*i*__, *usk*_*ID*_*i*__, *upk*_*ID*_*i*__〉 to *L*_*U*_ and returns *upk*_*ID*_*i*__ to *𝒜*1.
  - Otherwise (*ID*_*i*_ ≠ *ID*_*I*_), *𝒞* gets *Q*_*ID*_*i*__ = *a*_*ID*_*i*__ · *P* by making *H*_1_ query with *ID*_*i*_ and sets *psk*_*ID*_*i*__ = *a*_*ID*_*i*__ · *P*_*pub*_. *𝒞* selects a random number *x*_*ID*_*i*__ ∈ *Z*_q_* as his secret key *usk*_*ID*_*i*__ and computes his public key as *upk*_*ID*_*i*__ = *usk*_*ID*_*i*__ · *P*. At last, *𝒞* adds 〈*ID*_*i*_, *psk*_*ID*_*i*__, *Q*_*ID*_*i*__, *usk*_*ID*_*i*__, *upk*_*ID*_*i*__〉 to *L*_*U*_ and returns *upk*_*ID*_*i*__ to *𝒜*1.
- * RevealPartialKey*: when *𝒜*1 makes this query on *ID*_*i*_, *𝒞* does as follows.  
  - If *ID*_*i*_ = *ID*_*I*_, *𝒞* stops the simulation.
  - Otherwise (*ID*_*i*_ ≠ *ID*_*I*_), *𝒞* looks up the list *L*_*U*_ for the tuple 〈*ID*_*i*_, *psk*_*ID*_*i*__, *Q*_*ID*_*i*__, *usk*_*ID*_*i*__, *upk*_*ID*_*i*__〉 and returns *psk*_*ID*_*i*__ to *𝒜*1.
- * RevealSecertKey*: when *𝒜*1 makes this query on *ID*_*i*_, *𝒞* looks up the list *L*_*U*_ for the tuple 〈*ID*_*i*_, *psk*_*ID*_*i*__, *Q*_*ID*_*i*__, *usk*_*ID*_*i*__, *upk*_*ID*_*i*__〉 and returns *usk*_*ID*_*i*__ to *𝒜*1.
- * ReplaceKey*: when *𝒜*1 makes this query on (*ID*_*i*_, *upk*_*ID*_*i*__′), *𝒞* looks up the list *L*_*U*_ for the tuple 〈*ID*_*i*_, *psk*_*ID*_*i*__, *Q*_*ID*_*i*__, *usk*_*ID*_*i*__, *upk*_*ID*_*i*__〉. *𝒞* sets *usk*_*ID*_*i*__ ← ⊥ and replaces *upk*_*ID*_*i*__ with *upk*_*ID*_*i*__′.
- * Sign*: when *𝒜*1 makes this query on (*m*_*i*_, *ID*_*i*_), *𝒞* does as follows.
  - If *ID*_*i*_ = *ID*_*I*_, *𝒞* looks up lists *L*_*H*_1__ and *L*_*U*_ and for tuples 〈*ID*_*i*_, *a*_*ID*_*i*__, *Q*_*ID*_*i*__〉 and 〈*ID*_*i*_, *psk*_*ID*_*i*__, *Q*_*ID*_*i*__, *usk*_*ID*_*i*__, *upk*_*ID*_*i*__〉 separately, where *Q*_*ID*_*i*__ = *a*_*ID*_*i*__ *Y* and *upk*_*ID*_*i*__ may have been replaced by *𝒜*1. *𝒞* makes *H*_2_ query and *H*_3_ query with *params* and gets tuples 〈*params*, *b*_*i*_, *W*〉 and 〈*params*, *c*_*i*_, *T*〉, where *W* = *b*_*i*_ *P* and *T* = *c*_*i*_ *X*. *𝒞* generates three random numbers *h*_*i*_, *k*_*i*_, *r*_*i*_ ∈ *Z*_q_*, computes *U*_*i*_ = *k*_*i*_^−1^(*r*_*i*_ · *P* − *a*_*ID*_*i*__ · *c*_*i*_^−1^ · *Y*), *V*_*i*_ = *r*_*i*_ · *T* + *h*_*i*_ · *b*_*i*_ · *upk*_*ID*_*i*__, and sets *H*_4_(*m*_*i*_, *ID*_*i*_, *upk*_*ID*_*i*__, *U*_*i*_) ← *h*_*i*_, *H*_5_(*m*_*i*_, *ID*_*i*_, *upk*_*ID*_*i*__, *U*_*i*_) ← *k*_*i*_. At last, *𝒞* adds tuples 〈*m*_*i*_, *ID*_*i*_, *upk*_*ID*_*i*__, *U*_*i*_, *h*_*i*_〉 and 〈*m*_*i*_, *ID*_*i*_, *upk*_*ID*_*i*__, *U*_*i*_, *k*_*i*_〉 to *L*_*H*_4__ and *L*_*H*_5__ separately and returns (*U*_*i*_, *V*_*i*_) to *𝒜*1. It is easy to say (*U*_*i*_, *V*_*i*_) satisfies the equation *e*(*V*_*i*_, *P*) = *e*(*Q*_*ID*_*i*__, *P*_*pub*_)*e*(*h*_*i*_ · *upk*_*ID*_*i*__, *W*)*e*(*k*_*i*_ *U*_*i*_, *T*).
  - Otherwise (*ID*_*i*_ ≠ *ID*_*I*_), *𝒞* looks up lists *L*_*H*_1__ and *L*_*U*_ and for tuples 〈*ID*_*i*_, *a*_*ID*_*i*__, *Q*_*ID*_*i*__〉 and 〈*ID*_*i*_, *psk*_*ID*_*i*__, *Q*_*ID*_*i*__, *usk*_*ID*_*i*__, *upk*_*ID*_*i*__〉 separately, where *Q*_*ID*_*i*__ = *a*_*ID*_*i*__ *P* and *upk*_*ID*_*i*__ may have been replaced by (*𝒜*1). *𝒞* makes *H*_2_ query and *H*_3_ query with *params* and gets tuples 〈*params*, *b*_*i*_, *W*〉 and 〈*params*, *c*_*i*_, *T*〉, where *W* = *b*_*i*_ *P* and *T* = *c*_*i*_ *X*. *𝒞* generates a random number *r*_*i*_ ∈ *Z*_q_* and computes *U*_*i*_ = *r*_*i*_ *P*, *h*_*i*_ = *H*_4_(*m*_*i*_, *ID*_*i*_, *upk*_*ID*_*i*__, *U*_*i*_), *k*_*i*_ = *H*_5_(*m*_*i*_, *ID*_*i*_, *upk*_*ID*_*i*__, *U*_*i*_), and *V*_*i*_ = *psk*_*ID*_*i*__ + *h*_*i*_ · *b*_*i*_ · *upk*_*ID*_*i*__ + *k*_*i*_ · *r*_*i*_ · *T*. At last, *𝒞* returns (*U*_*i*_, *V*_*i*_) to *𝒜*1. It is easy to say (*U*_*i*_, *V*_*i*_) satisfies the equation *e*(*V*_*i*_, *P*) = *e*(*Q*_*ID*_*i*__, *P*_*pub*_)*e*(*h*_*i*_ · *upk*_*ID*_*i*__, *W*)*e*(*k*_*i*_ *U*_*i*_, *T*).

Finally, *𝒜*1 outputs a tuple (*ID*_*i*_, *m*_*i*_, *σ*_*i*_) as its forgery, where *σ*_*i*_ = (*U*_*i*_, *V*_*i*_). If *ID*_*i*_ ≠ *ID*_*I*_, *𝒞* stops the simulation. From the forgery lemma [27], if we have a replay of *𝒞* with the same random tape but different choice of *H*_4_ and *H*_5_, *𝒜*1 will output another three signatures. The following two equations hold since both of the two signatures are valid:

(5) $$\begin{array}{c} e\left(V_{i},P\right)=e\left(Q_{{ID}_{i}},P_{pub}\right)e\left(h_{i}\cdot upk_{{ID}_{i}},W\right)e\left(k_{i}U_{i},T\right),\\ e\left(V_{i}^{\prime },P\right)=e\left(Q_{{ID}_{i}},P_{pub}\right)e\left(h_{i}^{\prime }\cdot upk_{{ID}_{i}},W\right)e\left(k_{i}^{\prime }U_{i},T\right). \end{array}$$

*𝒞* looks up lists *L*_*H*_1__ and *L*_*U*_ and for tuples 〈*ID*_*i*_, *a*_*ID*_*i*__, *Q*_*ID*_*i*__〉 and 〈*ID*_*i*_, *psk*_*ID*_*i*__, *Q*_*ID*_*i*__, *usk*_*ID*_*i*__, *upk*_*ID*_*i*__〉 separately, where *Q*_*ID*_*i*__ = *a*_*ID*_*i*__ *P* and *upk*_*ID*_*i*__ may have been replaced by *𝒜*1. *𝒞* makes *H*_2_ query and *H*_3_ query with *params* and gets tuples 〈*params*, *b*_*i*_, *W*〉 and 〈*params*, *c*_*i*_, *T*〉, where *W* = *b*_*i*_ *P* and *T* = *c*_*i*_ *X*. At last, *𝒞* returns (*k*_*i*_(*V*_*i*_′ − *b*_*i*_ · *h*_*i*_′ · *upk*_*ID*_*i*__) − *k*_*i*_′(*V*_*i*_ − *b*_*i*_ · *h*_*i*_ · *upk*_*ID*_*i*__))/*a*_*ID*_*i*__(*k*_*i*_ − *k*_*i*_′) as the solution of CDH problem.

*Analysis*. We show that *𝒞* solves the given instance of the CDH problem with the probability *η*. To do so, we analyze the three events that result in *𝒞*'s success.

 
*E*_1_: *𝒞* does not abort in all the queries of *RevealPartialKey*.
 
*E*_2_: *𝒜*1 can forge a legal signature *σ*_*i*_ = (*U*_*i*_, *V*_*i*_).
 
*E*_3_: the outputted tuple (*ID*_*i*_, *m*_*i*_, *σ*_*i*_) satisfies *ID*_*i*_ = *ID*_*I*_.

From the simulation we know that Pr[*E*_1_] ≥ (1−(1/*q*_*H*_1__))^*q*_*RPK*_^, Pr[*E*_2_ | *E*_1_] ≥ *ε*, Pr[*E*_3_ | *E*_1_∧*E*_2_] ≥ (1/*q*_*H*_1__), where *q*_*H*_1__ and *q*_*RPK*_ denote the numbers of *H*_1_ queries and *RevealPartialKey* queries separately. Then, the probability that *𝒞* solves the CDH problem is

(6) η=Pr⁡[E1∧E2∧E3]=Pr[E1]Pr⁡[E2 ∣ E1]Pr[E3 ∣ E1∧E2]≥1qH1(1−1qH1)qEPPKε.

Then *𝒞* could solve the CDH problem with a nonnegligible probability since *ε* is nonnegligible. This contradicts the hardness of the CDH problem. Therefore, our CLS scheme is existentially unforgeable against Type I adversary in random oracle model under the assumption that the CDH problem is hard.

**Lemma 4**

If there is a Type I adversary *𝒜*2 wins Game 1 with nonnegligible probability *ε*. Then we could construct an algorithm *𝒞* to solve the CDH problem in *G*_1_ with nonnegligible probability.

**Proof**

Given an instance (*X* = *xP*, *Y* = *yP*) of the CDH problem in *G*_1_, we will construct an algorithm *𝒞* to compute *x* *yP*, where *x*, *y* ∈ *Z*_q_* and they are unknown to *𝒞*. At first, *𝒞* picks an identity *ID*_*I*_ at random as the challenged identity in this game, generates a random number *s* ∈ *Z*_q_* as the master key, sets the master public key *P*_*pub*_ = *sP*, selects the system parameters *params* = {*G*_1_, *G*_2_, *e*, *P*, *P*_*pub*_, *H*_1_, *H*_2_, *H*_3_, *H*_4_, *H*_5_}, and returns the master key and the parameters to *𝒜*2. Then *𝒞* answers *𝒜*2's query as follows.

- *H*_1_ *query*: *𝒞* maintains a list *L*_*H*_1__ of form 〈*ID*_*i*_, *a*_*ID*_*i*__, *Q*_*ID*_*i*__〉. When *𝒜*1 makes this query on *ID*_*i*_, *𝒞* does as follows.
  - If the list *L*_*H*_1__ contains a tuple 〈*ID*_*i*_, *a*_*ID*_*i*__, *Q*_*ID*_*i*__〉, *𝒞* returns *Q*_*ID*_*i*__ to *𝒜*2.
  - Otherwise, *𝒞* picks a random *a*_*ID*_*i*__ ∈ *Z*_n_*, computes *Q*_*ID*_*i*__ = *a*_*ID*_*i*__ *P*, adds 〈*ID*_*i*_, *a*_*ID*_*i*__, *Q*_*ID*_*i*__〉 to *L*_*H*_1__, and returns *Q*_*ID*_*i*__ to *𝒜*2.
- *H*_2_ *query: 𝒞* maintains a list *L*_*H*_2__ of form 〈*m*_*i*_, *b*_*i*_, *W*_*i*_〉. When *𝒜*2 makes this query on *m*_*i*_, *𝒞* does as follows.
  - If the list *L*_*H*_2__ contains a tuple 〈*m*_*i*_, *b*_*i*_, *W*_*i*_〉, *𝒞* returns *W*_*i*_ to *𝒜*2.
  - Otherwise, *𝒞* picks a random *b*_*i*_ ∈ *Z*_n_*, computes *W*_*i*_ = *b*_*i*_ *X*, adds 〈*m*_*i*_, *b*_*i*_, *W*_*i*_〉 to *L*_*H*_2__, and returns *W*_*i*_ to *𝒜*2.
- *H*_3_ *query*: *𝒞* maintains a list *L*_*H*_3__ of form 〈*m*_*i*_, *c*_*i*_, *T*_*i*_〉. When *𝒜*2 makes this query on *m*_*i*_, *𝒞* does as follows.   
  - If the list *L*_*H*_3__ contains a tuple 〈*m*_*i*_, *c*_*i*_, *T*_*i*_〉, *𝒞* returns *T*_*i*_ to *𝒜*2.
  - Otherwise, *𝒞* picks a random *c*_*i*_ ∈ *Z*_n_*, computes *T*_*i*_ = *c*_*i*_ *X*, adds 〈*m*_*i*_, *c*_*i*_, *T*_*i*_〉 to *L*_*H*_3__, and returns *T*_*i*_ to *𝒜*2.
- *H*_4_ *query*: *𝒞* maintains a list *L*_*H*_4__ of form 〈*m*_*i*_, *ID*_*i*_, *upk*_*ID*_*i*__, *U*_*i*_, *h*_*i*_〉. When *𝒜*2 makes this query on (*m*_*i*_, *ID*_*i*_, *upk*_*ID*_*i*__, *U*_*i*_), *𝒞* does as follows.
  - If the list *L*_*H*_4__ contains a tuple 〈*m*_*i*_, *ID*_*i*_, *upk*_*ID*_*i*__, *U*_*i*_, *h*_*i*_〉, *𝒞* returns *h*_*i*_ to *𝒜*2.
  - Otherwise, *𝒞* picks a random *h*_*i*_ ∈ *Z*_n_*, returns *h*_*i*_ to *𝒜*2, and adds 〈*m*_*i*_, *ID*_*i*_, *upk*_*ID*_*i*__, *U*_*i*_, *h*_*i*_〉 to *L*_*H*_4__.
- *H*_5_ *query*: *𝒞* maintains a list *L*_*H*_5__ of form 〈*m*_*i*_, *ID*_*i*_, *upk*_*ID*_*i*__, *U*_*i*_, *k*_*i*_〉. When *𝒜*2 makes this query on (*m*_*i*_, *ID*_*i*_, *upk*_*ID*_*i*__, *U*_*i*_), *𝒞* does as follows.   
  - If the list *L*_*H*_5__ contains a tuple 〈*m*_*i*_, *ID*_*i*_, *upk*_*ID*_*i*__, *U*_*i*_, *k*_*i*_〉, *𝒞* returns *k*_*i*_ to *𝒜*2.
  - Otherwise, *𝒞* picks a random *k*_*i*_ ∈ *Z*_n_*, returns *k*_*i*_ to *𝒜*2, and adds 〈*m*_*i*_, *ID*_*i*_, *upk*_*ID*_*i*__, *U*_*i*_, *h*_*i*_〉 to *L*_*H*_5__.
- * CreateUser*: *𝒞* maintains a list *L*_*U*_ of form 〈*ID*_*i*_, *psk*_*ID*_*i*__, *Q*_*ID*_*i*__, *usk*_*ID*_*i*__, *upk*_*ID*_*i*__〉. When *𝒜*2 makes this query on *ID*_*i*_, *𝒞* does as follows.
  - If the list *L*_*U*_ contains a tuple 〈*ID*_*i*_, *psk*_*ID*_*i*__, *Q*_*ID*_*i*__, *usk*_*ID*_*i*__, *upk*_*ID*_*i*__〉, *𝒞* returns *upk*_*ID*_*i*__ to *𝒜*2.
  - Otherwise, if *ID*_*i*_ = *ID*_*I*_, *𝒞* gets *Q*_*ID*_*I*__ = *a*_*ID*_*I*__ *P* by making *H*_1_ query with *ID*_*I*_ and computes *psk*_*ID*_*I*__ = *sQ*_*ID*_*I*__. *𝒞* sets *usk*_*ID*_*i*__ ← ⊥ and *upk*_*ID*_*i*__ ← *Y*. At last, *𝒞* adds 〈*ID*_*i*_, *psk*_*ID*_*i*__, *Q*_*ID*_*i*__, *usk*_*ID*_*i*__, *upk*_*ID*_*i*__〉 to *L*_*U*_ and returns *upk*_*ID*_*i*__ to *𝒜*2.
  - Otherwise (*ID*_*i*_ ≠ *ID*_*I*_), *𝒞* gets *Q*_*ID*_*i*__ = *a*_*ID*_*i*__ · *P* by making *H*_1_ query with *ID*_*i*_ and computes *psk*_*ID*_*I*__ = *sQ*_*ID*_*I*__. *𝒞* selects a random number *x*_*ID*_*i*__ ∈ *Z*_q_* as his secret key *usk*_*ID*_*i*__ and computes his public key as *upk*_*ID*_*i*__ = *usk*_*ID*_*i*__ · *P*. At last, *𝒞* adds 〈*ID*_*i*_, *psk*_*ID*_*i*__, *Q*_*ID*_*i*__, *usk*_*ID*_*i*__, *upk*_*ID*_*i*__〉 to *L*_*U*_ and returns *upk*_*ID*_*i*__ to *𝒜*1.
- * RevealPartialKey*: when *𝒜*2 makes this query on *ID*_*i*_, *𝒞* looks up the list *L*_*U*_ for the tuple 〈*ID*_*i*_, *psk*_*ID*_*i*__, *Q*_*ID*_*i*__, *usk*_*ID*_*i*__, *upk*_*ID*_*i*__〉 and returns *psk*_*ID*_*i*__ to *𝒜*2.
- * RevealSecertKey:* when *𝒜*2 makes this query on *ID*_*i*_, *𝒞* does as follows.
  - If *ID*_*i*_ = *ID*_*I*_, *𝒞* stops the simulation.
  - Otherwise (*ID*_*i*_ ≠ *ID*_*I*_), *𝒞* looks up the list *L*_*U*_ for the tuple 〈*ID*_*i*_, *psk*_*ID*_*i*__, *Q*_*ID*_*i*__, *usk*_*ID*_*i*__, *upk*_*ID*_*i*__〉 and returns *usk*_*ID*_*i*__ to *𝒜*2.
- * Sign*: when *𝒜*1 makes this query on (*m*_*i*_, *ID*_*i*_), *𝒞* does as follows.
  - If *ID*_*i*_ = *ID*_*I*_, *𝒞* looks up lists *L*_*H*_1__ and *L*_*U*_ and for tuples 〈*ID*_*i*_, *a*_*ID*_*i*__, *Q*_*ID*_*i*__〉 and 〈*ID*_*i*_, *psk*_*ID*_*i*__, *Q*_*ID*_*i*__, *usk*_*ID*_*i*__, *upk*_*ID*_*i*__〉 separately, where *Q*_*ID*_*i*__ = *a*_*ID*_*i*__ *P* and *upk*_*ID*_*i*__ may have been replaced by *𝒜*2. *𝒞* makes *H*_2_ query and *H*_3_ query with *params* and gets tuples 〈*params*, *b*_*i*_, *W*〉 and 〈*params*, *c*_*i*_, *T*〉, where *W* = *b*_*i*_ *X* and *T* = *c*_*i*_ *X*. *𝒞* generates three random numbers *h*_*i*_, *k*_*i*_, *r*_*i*_ ∈ *Z*_q_*, computes *U*_*i*_ = *k*_*i*_^−1^(*r*_*i*_ · *P* − *b*_*i*_ · *c*_*i*_^−1^ · *h*_*i*_ · *Y*), *V*_*i*_ = *r*_*i*_ · *T* + *a*_*ID*_*i*__ · *s* · *upk*_*ID*_*i*__, and sets *H*_4_(*m*_*i*_, *ID*_*i*_, *upk*_*ID*_*i*__, *U*_*i*_) ← *h*_*i*_, *H*_5_(*m*_*i*_, *ID*_*i*_, *upk*_*ID*_*i*__, *U*_*i*_) ← *k*_*i*_. At last, *𝒞* adds tuples 〈*m*_*i*_, *ID*_*i*_, *upk*_*ID*_*i*__, *U*_*i*_, *h*_*i*_〉 and 〈*m*_*i*_, *ID*_*i*_, *upk*_*ID*_*i*__, *U*_*i*_, *k*_*i*_〉 to *L*_*H*_4__ and *L*_*H*_5__ separately and returns (*U*_*i*_, *V*_*i*_) to *𝒜*2. It is easy to say (*U*_*i*_, *V*_*i*_) satisfies the equation *e*(*V*_*i*_, *P*) = *e*(*Q*_*ID*_*i*__, *P*_*pub*_)*e*(*h*_*i*_ · *upk*_*ID*_*i*__, *W*)*e*(*k*_*i*_ *U*_*i*_, *T*).
  - Otherwise (*ID*_*i*_ ≠ *ID*_*I*_), *𝒞* acts according to the description of the algorithm *Sign* since he knows both of *usk*_*ID*_*i*__ and *psk*_*ID*_*i*__.

Finally, *𝒜*2 outputs a tuple (*ID*_*i*_, *m*_*i*_, *σ*_*i*_) as its forgery, where *σ*_*i*_ = (*U*_*i*_, *V*_*i*_). If *ID*_*i*_ ≠ *ID*_*I*_, *𝒞* stops the simulation. From the forgery lemma [27], if we have a replay of *𝒞* with the same random tape but different choice of *H*_4_ and *H*_5_, *𝒜*2 will output another signatures. The following two equations hold since both of the two signatures are valid:

(7) $$\begin{array}{c} e\left(V_{i},P\right)=e\left(Q_{{ID}_{i}},P_{pub}\right)e\left(h_{i}\cdot upk_{{ID}_{i}},W\right)e\left(k_{i}U_{i},T\right),\\ e\left(V_{i}^{\prime },P\right)=e\left(Q_{{ID}_{i}},P_{pub}\right)e\left(h_{i}^{\prime }\cdot upk_{{ID}_{i}},W\right)e\left(k_{i}^{\prime }U_{i},T\right). \end{array}$$

*𝒞* looks up lists *L*_*H*_1__ and *L*_*U*_ and for tuples 〈*ID*_*i*_, *a*_*ID*_*i*__, *Q*_*ID*_*i*__〉 and 〈*ID*_*i*_, *psk*_*ID*_*i*__, *Q*_*ID*_*i*__, *usk*_*ID*_*i*__, *upk*_*ID*_*i*__〉 separately, where *Q*_*ID*_*i*__ = *a*_*ID*_*i*__ *P*. *𝒞* makes *H*_2_ query and *H*_3_ query with *params* and gets tuples 〈*params*, *b*_*i*_, *W*〉 and 〈*params*, *c*_*i*_, *T*〉, where *W* = *b*_*i*_ *X* and *T* = *c*_*i*_ *X*. At last, *𝒞* returns (*k*_*i*_(*V*_*i*_′ − *s* · *Q*_*ID*_*i*__) − *k*_*i*_′(*V*_*i*_ − *s* · *Q*_*ID*_*i*__))/*b*_*i*_(*h*_*i*_′*k*_*i*_ − *h*_*i*_ *k*_*i*_′) as the solution of CDH problem.

*Analysis*. We show that *𝒞* solves the given instance of the CDH problem with the probability *η*. To do so, we analyze the three events that result in *𝒞*'s success.

 
*E*_1_: *𝒞* does not abort in all the queries of *RevealSecertKey*.
 
*E*_2_: *𝒜*1 can forge a legal signature *σ*_*i*_ = (*U*_*i*_, *V*_*i*_).
 
*E*_3_: the outputted tuple (*ID*_*i*_, *m*_*i*_, *σ*_*i*_) satisfies *ID*_*i*_ = *ID*_*I*_.

From the simulation we know that Pr[*E*_1_]≥(1 − (1/*q*_*H*_1__))^*q*_*RSK*_^, Pr[*E*_2_ | *E*_1_] ≥ *ε*, Pr[*E*_3_ | *E*_1_∧*E*_2_] ≥ (1/*q*_*H*_1__), where *q*_*H*_1__ and *q*_*RSK*_ denote the numbers of *H*_1_ queries and *RevealSecertKey* queries separately. Then, the probability that *𝒞* solves the CDH problem is

(8) η=Pr⁡[E1∧E2∧E3]=Pr[E1]Pr⁡[E2 ∣ E1]Pr[E3 ∣ E1∧E2]≥1qH1(1−1qH1)qRSKε.

Then *𝒞* could solve the CDH problem with a nonnegligible probability since *ε* is nonnegligible. This contradicts the hardness of the CDH problem. Therefore, our CLS scheme is existentially unforgeable against a Type II adversary in random oracle model under the assumption that the CDH problem is hard.

From the above two lemmas, we could get the following theorem.

**Theorem 5**

The CLS scheme is secure against adaptively chosen warrant attacks and chosen message and identity attacks in the random oracle model if the CDH problem in *G*_1_ is intractable.

### 6.2. Security Analysis of Our CLAS Scheme

For the security of our CLAS scheme, we have the following theorem.

**Theorem 6**

The CLAS scheme is secure against adaptively chosen warrant attacks and chosen message and identity attacks in the random oracle model if the CDH problem in *G*_1_ is intractable.

**Proof**

Suppose there is an adversary *𝒜* ∈ {*𝒜*1, *𝒜*2} who could win Game 2 with nonnegligible probability. We could construct another adversary, who could win Game 1 with nonnegligible probability, through the method described in Theorem 2 of Xiong et al.'s work [25]. We have shown that no adversary could win Game 1 with nonnegligible probability in the above theorem. Therefore, our CLAS scheme is secure against adaptively chosen warrant attacks and chosen message and identity attacks in the random oracle model if the CDH problem in *G*_1_ is intractable.

## 7. Performance Analysis

In this section, we compare our scheme with two latest CLAS schemes, that is, Zhang et al.'s scheme [22] and Xiong et al.'s scheme [25]. For convenience, some notations are defined as follows:

- *T*_*P*_: the time for executing a pairing operation;
- *T*_*S*_: the time for executing a scalar multiplications in *G*_1_.

The comparisons are listed in Table 1. From the table, we know that Xiong et al.'s scheme and our scheme have better performance than Zhang et al.'s scheme. Xiong et al.'s scheme has better performance than our scheme. However, Xiong et al.'s scheme cannot withstand attacks of Type II adversary. It is well known that security is a top priority in network communications. It is acceptable to enhance security at the cost of increasing computational time slightly. Therefore, our scheme is more suitable for practical applications.

## 8. Conclusion

Recently, Xiong et al. proposed an efficient CLAS scheme. They claimed that both of their schemes are provably secure in the random oracle model. In this paper, we propose a new attack against their scheme. To overcome weakness, we also propose an improved CLAS scheme and show our scheme is provable in the random oracle model.

## Figures and Tables

**Table 1:** Performance comparisons.

	Sign	Verify	Aggregate verify
Zhang et al.'s scheme [22]	5*T*_*S*_	5*T*_*P*_ + 2*T*_*S*_	5*T*_*P*_ + 2*nT*_*S*_
Xiong et al.'s scheme [25]	3*T*_*S*_	3*T*_*P*_ + 2*T*_*S*_	3*T*_*P*_ + 2*nT*_*S*_
Our scheme	3*T*_*S*_	4*T*_*P*_ + 2*T*_*S*_	4*T*_*P*_ + 2*nT*_*S*_
